# Construct validity of the psychological general well being index (PGWBI) in a sample of patients undergoing treatment for stress-related exhaustion: a rasch analysis

**DOI:** 10.1186/1477-7525-11-2

**Published:** 2013-01-07

**Authors:** Åsa Lundgren-Nilsson, Ingibjörg H Jonsdottir, Gunnar Ahlborg, Alan Tennant

**Affiliations:** 1 The Sahlgrenska Academy, University of Gothenburg, Gothenburg, Sweden; 2 Institute of Stress Medicine (ISM), Gothenburg, Sweden; 3 Faculty of Medicine and Health, The University of Leeds, Leeds, United Kingdom; 4 Institute of Neuroscience and Physiology, Department of clinical neuroscience and rehabilitation, The Sahlgrenska Academy, University of Gothenburg, Per Dubbsgatan 14, plan 3, 413 45, Gothenburg, Sweden

**Keywords:** Rasch, Well-being, PGWBI, Stress

## Abstract

**Purpose:**

The Psychological General Well Being Index (PGWBI) is a widely used scale across many conditions. Over time issues have been raised about the dimensional structure of the scale, and it has not yet been subjected to scrutiny by modern Psychometric approaches. The current study thus evaluates the PGWBI with Rasch- and factor analysis.

**Methods:**

Consecutive patients recruited to a tertiary stress clinic were administered the PGBWI as part of routine clinical assessment at baseline and three months. Data from the scale was subjected to Factor Analyses and to Rasch analysis. In both cases adjustments for local independence violations were allowed.

**Results:**

179 patients were recruited, with a mean age of 43 years, and of whom 70% were female. An initial Confirmatory Factor Analysis (CFA) with baseline data failed, but the modification indices also indicated considerable levels of local dependency requiring errors to be correlated. An EFA highlighted positive and negative effect domains. Rasch analysis confirmed that fit of data to the model was influenced by local dependency, and that in practice if the items from the six underlying domains were treated as six ‘super’ items, the scale was shown to measure one dominant construct of well being. An interval scale transformation was therefore possible. A significant improvement in well-being was observed over a three month period.

**Conclusion:**

The PGWBI scale has satisfactory internal construct validity when tested with modern psychometric techniques, using data obtained from patients treated for stress-related exhaustion. The instrument has qualities that make it suitable also for monitoring well-being during interventions for stress-related exhaustion/clinical burnout.

## Introduction

Well-being is an important construct with a long history of use in health outcomes [1-3] and one of the unitary concepts which can be used to indicate quality of life [4]. Well-being has been shown to be an independent predictor of mortality and morbidity in different patient populations as well as in healthy populations [5,6]. Monitoring changes of perceived well-being and health in patient populations during treatment and rehabilitation is thus of great interest. Those with mental health problems as a consequence of prolonged psychosocial stress are one such population for which a more global measure of well-being, in addition to more specific clinical measures, may be valuable in determining the degree of recovery [7,8]. Dupoy’s scale, originally called the General Well-Being Schedule, and modified to the Psychological General Well-Being Index (PGWBI), has been widely used as such across many medical specialties and in many countries [9-15]. The scale comprises 22 polytomous items where a high score is indicative of high levels of psychological well-being. Six affective states are assessed within six subscales: anxiety, depressed mood, positive well-being, self-control, general health and vitality. Short versions of the scale have also been developed, as well as different scoring structures for items [16,17]. However, from time to time, psychometric analysis has questioned the factor structure of the original 22 item version, including the validity of its six underlying domains [18-20]. Also, no analysis of the structure of the scale has yet been undertaken with modern psychometric techniques. Consequently the current study revisits the scale from a factor analytic perspective and re-examines the construct validity of the PGWBI using Rasch analysis.

## Methods

### Participants

The present study consists of data from 179 patients that were referred from primary health care centers or occupational health service centers to a specialist clinic, which exclusively treats patients with stress-related mental disorders, in the region of Västra Götaland, Sweden. The patients were ambulatory at the time of the study, and none had received in-patient care due to their illness. The referral criteria were stress-related exhaustion with no apparent somatic disorder or abuse that could explain the exhaustion, and a maximum duration of ongoing sick leave of six months.

### Inclusion and exclusion criteria

The inclusion criteria for this study was 1) fulfilling the diagnostic criteria for Exhaustion Disorder (ED) [21] and 2) completed the multimodal treatment program (MMT) at the stress clinic. By fulfilling the inclusion criteria for ED, the patients should not have somatic diseases, such as generalised pain, thyroid disease or vitamin B-12 deficiency or obesity which could explain the exhaustion and these patients do not enter the treatment program. Also patients with alcohol abuse or serious psychiatric diagnoses other than depression and anxiety do not enter the treatment program at the clinic.

### Diagnostic and patients characteristics

The ED criteria were established by the Swedish National Board of Health and Welfare in 2003 to improve diagnostics in cases of stress-related exhaustion/clinical burnout and were assigned the code F43.8A of the International Classification of Diseases and Related Health Problems (ICD-10). The diagnostic procedure for ED has been described in detail elsewhere [22]. One important criterion requires that the physician, together with the patient, is able to identify one or more stressor that has been present for at least six months during which the symptoms developed. If the patient meets the criteria for major depressive disorder, dysthymic disorder or generalised anxiety disorder, these diagnoses are to be set first. Before consulting the physician, the patient completed a one-page Primary Care Evaluation of Mental Disorders symptom checklist. Affirmative responses were followed-up by the physician in a structured interview form conforming to the criteria of the Diagnostic and Statistical Manual of Mental Disorders, Fourth Revision, for the diagnostic assessment of mood and anxiety disorders (DSM-IV) [23]. These criteria have been previously described in several studies and are highly related to clinical burnout [22,24,25].

Patients that had taken part in an 18 month MMT program at the clinic between 2004 and 2008 were included in this study. The group considered in the current study consisted of 179 patients who had both baseline and 3 month assessments. The baseline characteristics for the patients included in the study are shown in Table 1. The majority of the patients remitted to clinic are women (70%).

**Table 1:** Descriptive data for the subjects included in the study (n = 179)

**Characteristics**	**Percent**
Women/Men (n = 125/54)	70/30
Percent with high educational level* (at least one year college education or more) (n = 179)	70
Percent scoring above >4.4 on burnout (SMBQ) (n = 173)	88
Percent with depression (clinical diagnoses) (n = 179)	79
Percent with anxiety (clinical diagnoses) (n = 179)	76
HAD Depression Score (n = 179)	
0-6	26
7-10	40
>10	34
HAD Anxiety Score (n = 178)	
0-6	10
7-10	26
>10	64

*HAD* Hospital and Anxiety depression scale.

*SMBQ* Shirom-Melamed burnout Questionnaire.

* High educational level is defined as having at least one year college education or more.

The MMT program includes similar component for all patients but are adapted to their individual needs, and the different component of the MMT program has been described in detail elsewhere [24].

#### Measurements

All patients entering the treatment program at the clinic were asked to fill in several questionnaires, including the PGWBI, both before and during treatment, and the current study focuses upon the PGWBI at baseline and at 3 months follow up. The Swedish version of the PGWBI was translated according to standard methodology, showing appropriate correlation with comparator measures (Nottingham Health profile and the Mood adjective Check list) [10,12]. As in the original, each item of the scale has five response options.

Various other measurements were used in the study to describe the patient group. These included the Shirom-Melamed Burnout Questionnaire (SMBQ) which includes 22 items with response scales graded from 1 (almost never) to 7 (almost always) [26,27]. A score above 4.4 on SMBQ in total score has previously been suggested to discriminate a clinical population from a non-clinic in regard to burnout [28]. The Hospital Anxiety and Depression Scale (HAD) was used to assess symptoms of depression and anxiety [29]. The HAD includes 14 items (7 items included in each sub-scale). Both subscales use the sum scores to classify “non-cases” (0-7), “possible cases” (8-10), and “cases” of depression and anxiety (>10) (Table 1).

### The rasch model

Data from the scale were fitted to the Rasch measurement model [30]. The purpose here is to see if the data satisfy, in a probabilistic manner, the axioms of additive conjoint measurement, and so conform to the requirements for effecting a transformation to interval scaling, rather than having to use the raw score of the scale, which is at the ordinal level [31-33]. This involves testing a series of assumptions, including the stochastic ordering of items, local response dependency, and unidimensionality [34]. Stochastic ordering is evaluated through fit to the model which reflects a probabilistic Guttman ordering [35]. A series of fit statistics are used to indicate adequacy of fit, and their ideal values are shown below at the bottom of the summary fit table (Table 2).

**Table 2:** Fit of PGWBI to the Rasch model

		**Item residual**		**Person residual**		**Chi square**		**PSI**	**Unidimensional test%(CI)**
**Analysis name**	**# of items**	**Mean**	**±SD**	**Mean**	**±SD**	**Value**	** p**		
1. Pos Well Being	4	-0.24	0.96	-0.42	0.84	27.9	0.68	0.81	5.59 (3.8-11.2)
2. General Health	3	0.42	0.92	-0.40	0.95	12.6	0.18	0.59	1.12 (-0.2-4.3)
3. Depressed Mood	3	-0.06	0.67	-0.40	0.89	21.4	0.77	0.88	5.03 (1.8-8.2)
4. Self Control	3	0.21	0.34	-0.30	0.77	9.5	0.40	0.65	2.79 (0.0-6.0)
5. Anxiety	5	0.28	1.74	-0.41	1.15	51.8	<0.00	0.81	7.26 (4.1-10.5)
6. Anxiety	3	-0.10	1.66	-0.58	1.19	15.8	0.08	0.72	3.37 (0.2-6.5)
7. Vitality	4	0.26	1.39	-0.38	0.87	9.89	0.62	0.85	3.91 (0.7-7.1)
8. Time 1	22	0.58	2.39	-0.15	1.55	158.5	< 0.01	0.92	25.7 (22.5-28.9)
9. Six testlet	6 (22)	0.25	1.78	-0.35	1.15	18.3	0.11	0.85	7.8 (4.6-11.0)
10. Time 2	22	0.30	3.01	-0.13	1.59	399.2	<0.01	0.94	21.2 (18.0-24.4)
11. Six testlet 2	3 (22)	0.21	2.23	-0.36	1.21	22.5	0.03	0.89	8.9 (5.7-12.1)
12. Five testlet 2	5 (22)	0.12	2.11	-0.42	1.23	20.1	0.17	0.88	7.3 (4.1-10.5)
13. Pooled	2 (22)	0.14	0.92	-0.64	0.99	3.8	0.95	0.86	4.9 (2.6-7.1)
***Ideal Values***		***0.0***	***<1.4***^***a***^	***0.0***	***<1.4***		***>0.05***^***b***^	***>0.85***	***(LCI <5%)***

^a)^ May be higher when unequal length testlets present ^b)^ Bonferroni adjusted.

The item trait interaction and standardized mean person and item fit, was evaluated by using *X*^*2*^ statistics with non-significant *X*^*2*^ probability values. A significant *X*^*2*^ indicates that the hierarchical ordering of the items varies across the trait being measured (ie, psychological well-being), which compromises the required property of invariance. Available as a summary fit statistic, and for each individual item, Bonferroni corrections are applied to the *X*^*2*^ at the 0.05 level.

The standardized mean values of the summary person and item fit residuals by a mean (SD) score of 0.0 ± 1.0 indicates perfect fit. At the individual item-and person level of fit, a nonsignificant *X*^*2*^ probability value and standardized fit residuals of between -2.5 and +2.5 indicate adequate fit the latter consistent with the 99% confidence interval for the residuals, thus allowing for some recognition of multiple testing (i.e. setting the significance level at 0.01).

Local response dependency is where items are linked in some way, for example two items about climbing stairs, where one asks about difficulty for climbing a single flight, the second about several flights. If a respondent has no difficulty in climbing several flights of stairs, then they must also have no difficulty climbing a single flight of stairs. This breaches the local independence assumption that says that, conditioning on the trait being measured, responses to items must be independent [36]. The presence of local dependency inflates reliability, and compromises parameter estimation [37]. Local response dependency can be identified through the correlation of residuals which, in the current analysis, is a value of 0.2 above the average residual correlation. The problem can be accommodated though testlets where the items are simply summed together into a ‘super item’ or testlet (in the climbing stairs example this would form the equivalent of one question asking how many flights of stairs can be climbed without difficulty) [38]. Where all items are reduced to a set of testlets this is formally equivalent to a bi-factor model [39]. The latent correlation between testlets can also be determined, as well as the proportion of non-error variance accounted for when the testlets (super items) are added together to make a total score [40].

As a basic assumption of summating any set of items to make a total score is that the set are unidimensional, it is crucial to ensure that this is the case [41]. In RUMM2030, the software used in the current study, Smith’s test of unidimensionality is implemented whereby items loading positively and negatively on the first principal component of the residuals are used to make two independent person estimates (in this case of well-being), and these are contrasted through a series of independent t-tests [42]. Person estimates from these subtests were compared, and if more than 5% of these tests were found to be significant, then the scale was considered multidimensional.

A binomial confidence interval of proportions can be used to show that the lower confidence interval of the observed proportion falls below the 5% level.

In addition the process of Rasch analysis also allows for an investigation of polytomous item threshold ordering and Differential Item Functioning (DIF). Threshold ordering is important to ensure that the increase in the category of response to an item, represented by the transition point (threshold) between categories, reflects an increase in the underlying trait. Where this fails, it is indicative of a ‘disordered threshold’, which can be adjusted by the collapsing of categories.

For DIF the response to an item, condition upon the level of the trait, should not differ across group membership such as gender. When this is found to differ, it can be dealt with by ‘splitting’ items such that, for example, an item becomes two items, one for each gender, with structural missing values for the excluded gender. In this paper DIF by age, gender, and whether or not the patient was working, was tested.

A reliability index (Person Separation Index - PSI) is also reported. Where data are normally distributed this can be interpreted as similar to Cronbach’s alpha, and thus values of 0.7 and 0.85 are indicative of reliability sufficient for group and individual use respectively [43]. Where the distribution of data departs from normality, it is useful to view both the PSI and alpha, to gain insight into the effect of skewness and floor and ceiling effects. Under these circumstances the PSI reflects the number of statistically significant groups of patients (strata) that can be identified by the instrument [44].

Data from each time point was initially separately analysed. Once data were shown to fit the Rasch model, the data was pooled and tested for invariance (lack of DIF) over time. The procedure by Mallinson was used to accommodate repeated measures [45]. Given fit to the Rasch model, an interval scale latent estimate (in logits) is available for further analysis, with the raw score transformed into a suitable range, for example 0-100.

Targeting of persons and items (person-item threshold distribution) was made by comparing the mean location score obtained for the patients with that of the items (almost always zero at the center of the scale). In the Rasch model, the center of the scale represents the item (in the dichotomous case) of average difficulty [46].

The data from this scale were also subjected to a Confirmatory Factor Analysis (CFA) to gain insight into both the comparison with the Rasch analysis, and previous published factor analysis of the scale. In the case where the scale fails the CFA (where the correlation of error terms would be allowed), an Exploratory Factor Analysis (EFA) with a PROMAX rotation would be considered. The Root Mean Square Error of Approximation (RMSEA) is reported here (where a value of <0.10 is considered weak, and a value of <0.08 is considered a moderately supported of a unidimensional structure; and additional statistics including the Tucker Lewis Index (TLI) and the Comparative Fit Index (CFI) are indicative of a unidimensional construct when their values exceed 0.95.

Mplus 6 was used for the CFA and EFA [47], and RUMM 2030 for the Rasch analysis [48]. All other analysis was undertaken with SPSS18 [49].

The study was approved by The Regional Ethical Review Board in Gothenburg (243-05) and conduced in compliance with the Helsinki declaration.

## Results

179 patients (125 women – 70%) with a median age 43 were assessed at 0 and 3 months. Median levels of PGWB were 73.0 (IQR 62.0-80.0) and 86.0 (IQR 74.0-98.0), respectively. 88% of the patients also fulfilled the criteria for clinical burnout (defined as scoring above 4.4 on the Shirom-Melamed burnout questionnaire at baseline).

Initially, for comparison with earlier work, a confirmatory factor analysis on the total 22 items failed to support a total score (CFI = 0.894; TLI = 0.883; RMSEA 0.145). However, modification indices indicated considerable local dependency in the data, requiring correlation of error terms. Where the data are treated as six items (the sum of items within each domain – effectively item parceling based upon the underlying conceptual structure), then, allowing for correlated errors, the CFA is satisfactory (CFI; TLI >0.95; Chi-square 6.4 (7df) p = 0.49). An Exploratory Factor Analysis on the 22 items also indicated a two-factor solution with mediocre fit (RMSEA 0.095) and reflected the positive- and negative affect sets of items.

Items from each individual subscale were then fitted to the Rasch model using the unrestricted model (partial credit model). All but the anxiety subscales satisfied fit to the model, with invariance (no DIF) across age, gender, and whether or not the patient was working or not, and satisfying the local independence and unidimensionality assumptions (Table 2, Analyses 1-7). The anxiety subscale required adjustment for local dependency between two pairs of items (made into a testlet), and then satisfied all requirements (Analysis 6).

Initial fit of the 22 item scale to the Rasch model was poor (Table 1, Analysis 8). The majority of items displayed ordered thresholds, and in the two items that did not, two categories were collapsed before testlets were created. Of note, no item showed DIF by age, gender, and whether or not the patient was working, or not. Reliability (PSI) was also high at 0.92. However, multidimensionality was observed and, importantly, as with the CFA, considerable local response dependency was observed within the cluster of items belonging to each of the six subscales. Given this observed local dependency, the items were made into six testlets (domain scores) (which would be added together to give the overall score, and which is consistent with the scoring instructions given in the manual). Fit improved considerably, and the data were unidimensional (Analysis 9). The average latent correlation between the six testlets was 0.65, and when all six were added together to make a total score, 93% of the total non-error variance was found to be common. This supports the hypothesis that the respondents profiles on the six subscales could be summarized by a single number, which is further evidence of the unidimensionality complementing the post-hoc test which showed 7.8% (CI: 4.6-11.0) of estimates to vary.

This solution was tested on the data from the second time point. Initially, for all 22 items, fit to the model was again poor, and multidimensionality was evident (Analysis 10). Once again the data were merged into six testlets with fit to the model but, on this occasion, some residual local dependency and marginal multidimensionality was observed (Analysis 11). The ‘positive well-being’ and ‘depressed mood’ testlets showed further local dependency, and these were merged, so making five testlets in all. These data then fully accorded with all assumptions of the model (Analysis 12).

Data from both time points were subsequently pooled and, following implementation of the recommendations by Mallinson to accommodate repeated measures, were made into two testlets representing positive and negative affect to accommodate local dependency in the items (i.e. the analysis of the residuals was consistent with the earlier Factor analysis). The data demonstrated fit to the model, unidimensionality and no DIF was observed by time (Analysis 13). The latent correlation between the positive and negative affect testlets was 0.90, and when the two are added together to give a total score, 96% of total non-error variance was common, again supporting a single construct of psychological well-being, and complementary to the 4.9% of t-tests (CI: 2.6-7.1) which supported the unidimensionality of the instrument.

**Figure 1:**
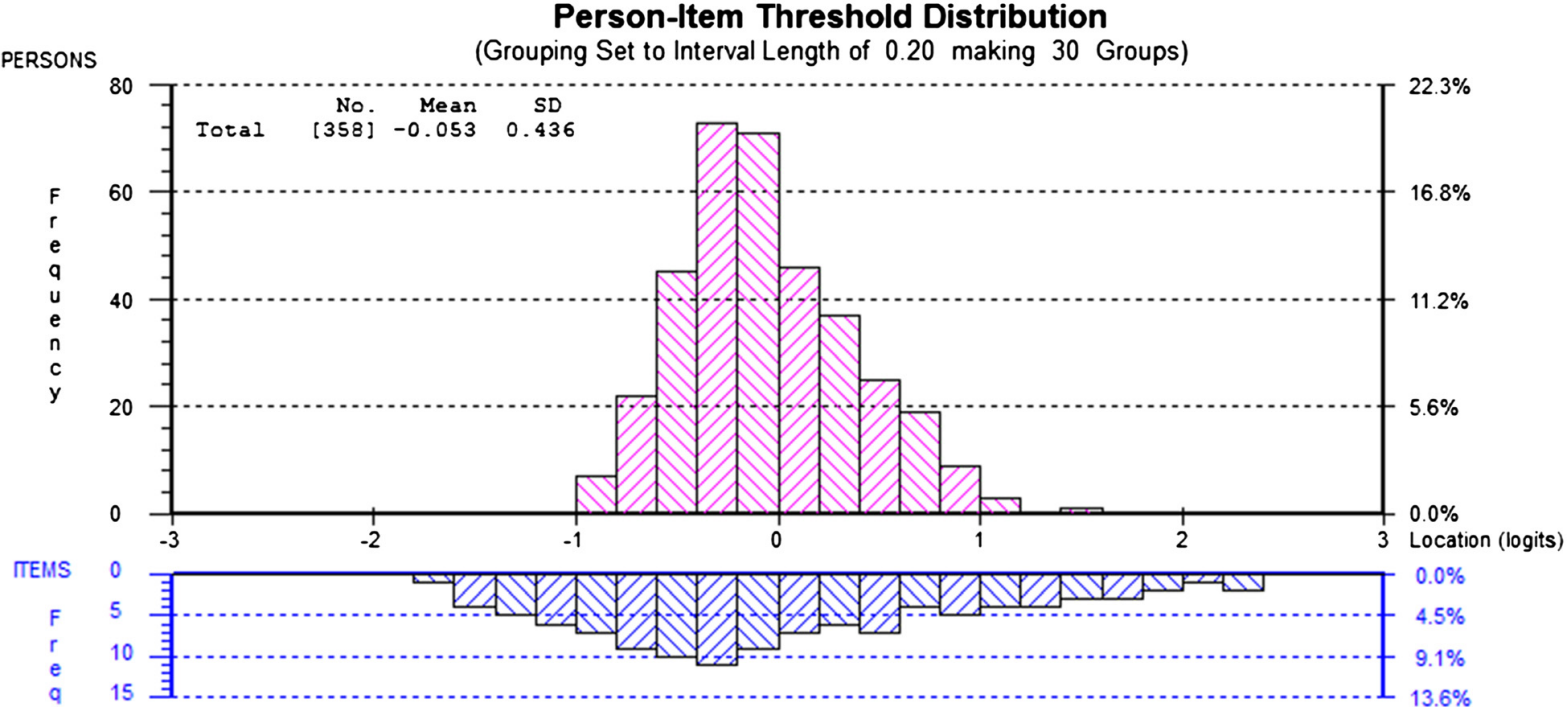
Targeting of persons and items.

The scale was almost perfectly targeted to the clinical sample (analysis 13), with the mean of persons being -0.053 on the logit scale (given the scale itself is centred at zero logits) (Figure 1). There was no significant difference in well-being by either age or gender (DIF) (p >0.05) in the pooled data. However, a significant improvement in well-being in logits was observed over the three months of the two assessments (F27.9; p < 0.001). The metric based effect size was 1.152.

Given fit to the Rasch model with all 22 items (presented as two testlets) a raw score to interval scale conversion table is available (Table 3). This gives two raw scores, depending upon whether or not the items are scored in the traditional 1-6 mode (so giving a range of 22-132), or as 0-5 (range 0-110), together with the metric conversion. This transformation is valid when all data are present. To use this table, simply sum the responses to all items and look up the metric equivalent to your raw score. The latent metric estimate has been adjusted to remove the unique variance (4%) in the data.

**Table 3:** Transformation of raw score to metric

**1-6**	**0-5**	**Metric**
22	0	0.0
23	1	7.6
24	2	12.1
25	3	14.9
26	4	16.8
27	5	18.3
28	6	19.6
29	7	20.7
30	8	21.7
31	9	22.5
32	10	23.3
33	11	24.0
34	12	24.7
35	13	25.4
36	14	26.0
37	15	26.6
38	16	27.1
39	17	27.7
40	18	28.2
41	19	28.7
42	20	29.2
43	21	29.7
44	22	30.1
45	23	30.6
46	24	31.0
47	25	31.5
48	26	31.9
49	27	32.3
50	28	32.7
51	29	33.1
52	30	33.5
53	31	33.9
54	32	34.3
55	33	34.7
56	34	35.0
57	35	35.4
58	36	35.8
59	37	36.1
60	38	36.5
61	39	36.8
62	40	37.2
63	41	37.5
64	42	37.9
65	43	38.2
66	44	38.6
67	45	38.9
68	46	39.3
69	47	39.6
70	48	40.0
71	49	40.4
72	50	40.7
73	51	41.1
74	52	41.5
75	53	41.9
76	54	42.3
77	55	42.6
78	56	43.1
79	57	43.4
80	58	43.9
81	59	44.3
82	60	44.7
83	61	45.1
84	62	45.6
85	63	46.0
86	64	46.5
87	65	46.9
88	66	47.4
89	67	47.9
90	68	48.4
91	69	48.9
92	70	49.4
93	71	49.9
94	72	50.5
95	73	51.0
96	74	51.6
97	75	52.1
98	76	52.7
99	77	53.3
100	78	53.9
101	79	54.5
102	80	55.2
103	81	55.8
104	82	56.5
105	83	57.2
106	84	57.9
107	85	58.5
108	86	59.3
109	87	60.0
110	88	60.8
111	89	61.6
112	90	62.4
113	91	63.2
114	92	64.0
115	93	64.9
116	94	65.8
117	95	66.8
118	96	67.8
119	97	68.8
120	98	69.9
121	99	71.0
122	100	72.3
123	101	73.5
124	102	74.9
125	103	76.4
126	104	78.0
127	105	79.8
128	106	81.8
129	107	84.3
130	108	87.4
131	109	92.4
132	110	100.0

## Discussion

Using data derived from patients undergoing treatment for stress-related exhaustion/burnout, the current study has that, for the modern psychometric perspective of Rasch analysis, the PGWBI satisfies model expectations at both the individual subscale level, and the 22 item level, having accommodated local response dependency where necessary. Thus the summed score, for both subscales and the total score is valid, and can be transformed into an interval scale derived from the latent estimate. The total score reflects the scoring structure indicated in the manual, that is the six domains are summed, and then the domain totals are summed together to make the total score. The testlet solution used in the current study has been shown to be formally equivalent to the bi-factor model [39,50]. The high latent correlations between the various testlet designs (e.g. positive and negative affect) and the high common variance found, suggests that a total score summarizes the well-being profile of the majority of persons. A CFA of this approach (i.e. six items as an item parcel design) also supported the total score solution when local dependency was accommodated.

The study has a number of implications. The PGWBI showed a considerable effect size over a three month treatment period, suggesting it may be a responsive instrument for studies associated with stress and burnout, and so measure the impact of interventions upon well-being and quality of life. On this occasion the metric based effect size was higher than that based on the ordinal data (0.97), reflecting the bias introduced by patients moving across the margins of the scale. Here, raw score points understate the true magnitude of change, whereas in the middle of the scale, they over emphasise the magnitude of change. Given the effect size formulae involves mathematical calculations, requiring interval level data, then the metric version is correct [33].

The interval scale latent estimate also opens up the possibility of more sophisticated models to examine the impact of mediators and effect modifiers upon well-being in the context of work-related stress. Rasch-transformed interval scale single indicator latent estimates can be included in such models, having dealt with bias caused by local dependency or Differential Item Function, and perhaps most important, missing values (the latent estimate is based upon the information available). Such indictors may be modeled over time, for example within a latent growth curve model, so as to understand trajectories of change [51].

The study also raises questions about factor analytic interpretation in the presence of local dependency. Given the cluster of items within each of the underlying six domains of the scale (e.g. positive well being; depressive symptoms) and their observed level of local dependency, it is possible that some of the variability in earlier factor analytic findings may have been influenced by this local dependency, causing CFA to fail, and perhaps generating spurious factors. The current study supports a strong single construct of psychological well being, once this dependency is accommodated, both from a Rasch analysis perspective, and a factor analytic perspective.

Limitations should also be considered. The patient population included in the study comprised a selected group of more severe cases of stress-related exhaustion/burnout remitted for specialist evaluation and treatment. They scored on average lower on the PGWBI compared to several other patient groups with severe health problems e.g. waiting for coronary bypass surgery [52] and gastroesophageal reflux disease [53]. Thus, most of them started from a very low level of psychological well-being and, in spite of the considerable improvement over the first three months of treatment, still scored much below expected from a healthy population at the second measurement. Populations with less pronounced stress-related health problems would be expected to show higher PGWBI scores both before and after intervention of similar duration, and invariance of the scale across such severity groups will need to be demonstrated. If the effect size for such an intervention had been calculated inappropriately on ordinal data, then its magnitude may have been smaller (as was shown to be the case in the current study) due to the misuse of ordinal raw scores in mathematical calculations [54]. Given this bias is greatest at the margins of the scale it is possible that traditional effect size calculations associated with this scale may have considerably underestimated its value when associated with those with very low levels of well-being upon entry into treatment, or over estimated its magnitude when patients were entering and exiting treatment over the central part of the scale. The transformation table provided is useful for researchers who wish to use interval-level data but cannot or do not want to perform their own Rasch analysis. In such cases, researchers can simply calculate the summary score as normal then use the table provided to convert this raw score into a latent estimate at the interval-level of measurement.

In conclusion, we found the PGWBI instrument to have satisfactory internal construct validity when tested with modern psychometric techniques, using data obtained from patients treated for stress-related exhaustion. Both individual domains as well as a total score are valid, given accommodation for local dependency. However, the transformation table simply requires a summed score from the questionnaire, as it is the latent estimate that has been adjusted for these effects. The instrument has been shown to possess qualities, including reliability sufficient for individual use, that make it suitable for monitoring well-being during interventions for stress-related exhaustion/clinical burnout.

## Abbreviations

PGWBI: Psychological General Well-Being Index; EFA: Exploratory Factor Analyses; CFA: Confirmatory Factor Analyses; RMSEA: Root Mean Square Error of Approximation; DIF: Differential Item Functioning; IQR: Inter quartile range; CFI: Comparative fit index; TLI: Tucker-Lewis index; PSI: Person separation index.

## Competing interests

The authors declare that they have no competing interests.

## Authors’ contributions

ÅLN analyzed the data for this study and contributed to the writing of the manuscript. AT analyzed the data for this study and contributed to the writing of the manuscript. IJ gathered the data for this study and contributed to the writing of the manuscript. GA Jr gathered the data for this study and contributed to the writing of the manuscript. All authors read and approved the final manuscript.
